# Busyness, mental engagement, and stress: Relationships to neurocognitive aging and behavior

**DOI:** 10.3389/fnagi.2022.980599

**Published:** 2022-08-24

**Authors:** Sara B. Festini

**Affiliations:** Department of Psychology, University of Tampa, Tampa, FL, United States

**Keywords:** busyness, cognitive reserve, aging, stress, cognition, daily activities, memory

## Abstract

Considerable research identifies benefits of sustaining mental engagement in older adulthood. Frequent social, mental, and physical activities (e.g., exercise) and lifestyle factors that bolster cognitive reserve (i.e., education, occupation complexity) have been associated with cognitive benefits and delayed onset of dementia. Nevertheless, the relationship between general daily levels of busyness and cognition has been relatively understudied. Open questions remain about whether a causal link exists between a busy lifestyle and mental prowess, the relationship between busyness and stress, and methodological approaches to measure and track busyness levels. Here, the existing evidence is considered, along with future directions for research aimed at characterizing the effects of a busy lifestyle on neurocognitive aging and behavior.

## Introduction

Extensive evidence indicates that cognition shows deficits with increasing age (see Salthouse, 2009; Park and Festini, 2017, 2018). In particular, fluid cognitive abilities like processing speed and episodic memory show the largest age-related decrements, whereas crystallized abilities like semantic memory (e.g., vocabulary) remain stable or increase with age (Park et al., 2002). Age-related diseases, like Alzheimer’s and cerebrovascular disease, also contribute to age-related cognitive deficits, and it can be difficult to differentiate non-pathological aging from underlying pre-clinical disease processes (Sperling et al., 2011). To counteract cognitive deficits associated with advanced age, some research has aimed to identify sources of cognitive preservation in older adulthood. Multiple studies have documented benefits of social, mental, and physical activities (i.e., exercise; new learning) on cognition and brain health in older adults (e.g., Colcombe et al., 2006; Carlson et al., 2009; Park et al., 2014). Further, lifestyle factors that promote cognitive reserve (Stern, 2002), such as high education and occupation complexity (see Hussenoeder et al., 2019) have been found to be related with better cognitive functioning (e.g., Correa Ribeiro et al., 2013) and lower risk of Alzheimer’s disease (e.g., Andel et al., 2005).

However, relatively little research has been conducted to examine the effects of general daily levels of busyness on cognition. Busyness can be defined as having one’s time occupied by frequent obligations (Gershuny, 2005). The Martin and Park Environmental Demands Questionnaire (MPED; Martin and Park, 2003) busyness subscale has been used as an assessment of busyness (e.g., Festini et al., 2016, 2019; Kaya et al., 2019). This self-report busyness subscale includes questions like, “*How often do you have too many things to do each day to actually get them all done?”* or “*How often do you rush out of the house in the mornings to get where you need to be?*” Thus, it is distinct from other measures of mental engagement and stress because it specifically assesses busyness and task load. Festini et al. (2016) reported that busier middle-aged and older adults tended to have better cognition, with the strongest relationship observed for episodic memory. This provided an initial demonstration of the potential benefits of living a busy, engaged lifestyle, but much additional research needs to be conducted. It is still unknown whether this relationship is causal–that is, whether or not being busy causes preservation of cognitive abilities, or if the relationship was observed simply because people with better mental function are capable of living busier lives. Moreover, it will be imperative to examine the interaction between busyness, cognition, and stress within the same individuals because it is possible that busyness that becomes stressful may impair cognitive performance, as literature also frequently observes negative consequences of stress on cognitive and brain health (Lupien et al., 2009). Here, I address several important areas for future research, while situating these future studies in the current literature. The focus is primarily on psychological research that addresses “busyness” and “busy lifestyles” directly. Important relevant literature on related concepts regarding mental engagement is briefly considered (for comprehensive reviews see Butler et al., 2018; Bielak and Gow, 2022; Roheger et al., 2022).

## Brief review of critical literature

### Activity levels

Substantial correlational evidence documents a relationship between heightened activity levels and better cognitive and neural health. Activity level research often implements self-report assessments of a broad range of daily activities, including how often individuals partake in social, physical, and cognitive activities. For example, Seeman et al. (2011) observed that greater social engagement was associated with better episodic memory and executive functioning in middle-aged and older adults. Similarly, Valenzuela and Sachdev (2007) found that individuals with more lifetime experiences, an assessment of intellectual activity across one’s lifetime, had less cognitive decline over 18 months. Activity level research has repeatedly documented favorable associations between frequent activities and neurocognitive aging (see Anaturk et al., 2018; Gheysen et al., 2018).

### Cognitive reserve, brain maintenance, and STAC-r

The related concepts of cognitive reserve and brain maintenance propose that characteristics like education, occupation complexity, and intellectual challenge can promote maintenance of cognitive function despite brain pathology (e.g., Stern, 2002; Barulli and Stern, 2013; Stern et al., 2020). That is, certain lifestyle factors are proposed to be protective that allow older adults to maintain better overall cognition and delay symptoms of cognitive decline (Soldan et al., 2017; for a review see Song et al., 2022). For example, those individuals with higher education showed more brain atrophy, despite similar cognitive performance (Coffey et al., 1999), suggesting that higher education enables preservation of cognitive faculties despite more pronounced brain pathology. When examining a composite measure of cognitive reserve that included education, occupation, IQ, and intellectual/social activities, Sole-Padulles et al. (2009) observed that cognitive reserve was associated with both larger brain size and increased neural efficiency (cf. Foubert-Samier et al., 2012).

Also considering protective neural enrichment factors, the Scaffolding Theory of Aging and Cognition-revised (STAC-r; Reuter-Lorenz and Park, 2014) proposes that lifestyle factors like education, physical fitness, and multilingualism can promote compensatory neural scaffolding that assists performance. Older adults who have better brain health and who more efficiently use alternate neural resources are proposed to exhibit better cognition and less cognitive decline (see Festini et al., 2018).

### Stress and cognition

Much prior research has documented the detrimental effects of stress on neurocognitive function. Non-human animal studies display that unpredictable chronic stress can impair memory, increase anxiety and depressive symptoms, as well as reduce the growth of new neurons in the hippocampus (see Parihar et al., 2011). For instance, Li et al. (2008) exposed mice to chronic mild stress, such as periods of restricted access to food or continuous light, for 5 weeks, and observed memory disruption. Chen et al. (2010) found that even a 5-h period of acute stress impaired memory, reduced hippocampal dendritic spine density, and disrupted long-term potentiation in mice. Similarly, in humans, stress impairs mental functioning under certain contexts. Oei et al. (2006) observed that stress impaired human working memory performance at high memory loads only (for a review see Martin et al., 2019). The stress hormone cortisol has also been shown to impair memory retrieval of well-learned memories in humans (Wolf et al., 2004). And, literature on burnout finds that uncontrollable stress and feeling over-worked can disrupt not only cognitive performance but also interpersonal interactions and wellbeing (Arnsten and Shanafelt, 2021; Romito et al., 2021).

Nevertheless, some studies report benefits of mild stress. Kofman et al. (2006) observed that undergraduates exhibited superior task-switching and attentional control when anxiety levels were higher at the end-of-the-semester. Some studies also observed that prolonged mild stress can increase neurogenesis in the hippocampus, improve memory, and reduce symptoms of depression and anxiety in rats (Parihar et al., 2011).

Thus, it has been proposed that the relationship between stress and cognition follows an inverted-U pattern (Lupien et al., 2009), such that optimal performance occurs with moderate stress, but that too little or too much stress impairs performance (cf. Yerkes and Dodson, 1908). Perhaps the relationship between busyness and mental functioning follows a similar pattern.

### Current research on busyness

Festini et al. (2016) examined the relationship between busyness and cognition in middle-aged and older adults. Those participants who were busier tended to have better processing speed, working memory, reasoning, and crystallized knowledge, with the strongest correlation between busyness and episodic memory. Moreover, busyness accounted for additional variance in all cognitive constructs, even after controlling for age and education.

Notably, these effect sizes between busyness and cognition were small to moderate (magnitudes of 0.16 to 0.32). These effects were observed with a relatively large sample size (330 participants). Thus, in future research, although cumbersome, relatively large samples will be needed to have sufficient power to detect such effect sizes.

Additional busyness research has observed that many, but not all, individuals perceive themselves as busy. Kaya et al. (2019) reported that over three-quarters of their sample of 22- to 54-year-olds characterized themselves as a busy person. Moreover, being busy has been proposed to be a badge of honor, demonstrating high social status and frequent contributions toward society (Gershuny, 2005; Bellezza et al., 2016). Relatedly, research on time shortage perceptions indicates that people often report feeling that they do not have sufficient time to complete what they want to do and feel rushed (Rudd, 2019). A model that considered demographic, personality, health, and activity measures found that busyness was best predicted by younger age, female gender, agreeable and neurotic personality, high levels of need for cognition (i.e., enjoyment of effortful thinking; Cacioppo et al., 1984), and frequent participation in novel activities (Festini et al., 2019).

Related research on retirement has found that partial retirement in the same job negatively impacted cognition, whereas partial retirement with a new employer benefited cognition in those with complex occupations (Mizuochi and Raymo, 2022; cf. Kajitani et al., 2016). This is consistent with the notion that busyness levels drop during retirement, and that new learning at a novel workplace is beneficial to cognition. Interestingly, Atalay et al. (2019) observed similar cognitive decline following retirement, regardless of whether it was forced or voluntary, suggesting that, indeed, the reduction in mental engagement contributes to cognitive decline.

## Areas for future research on busyness

### Research targeting a causal question

Although methodologically difficult, experimentally manipulating busyness levels is needed to address causality. Currently, only correlational assessments between busyness and cognitive abilities have been performed due to the difficulty of randomly assigning busyness.^1^ Nevertheless, lifestyle interventions have been conducted previously with success. See Table 1 for example intervention studies and their observed benefits (see also Butler et al., 2018; Gomes-Osman et al., 2018). This experimental evidence provides support for use-dependent neural plasticity (e.g., May, 2011; McDonough et al., 2015) and offers a proposed mechanism for why cognition can improve with sustained mental challenge.

**TABLE 1:** Example lifestyle interventions and their impacts on cognitive health.

Intervention	Citation	Method	Benefits
Synapse project	Park et al. (2014)	New learning: Digital photography, quilting, or both vs. active or passive control groups	Better episodic memory
		14 weeks	
		60–90 yrs	
Synapse project fMRI	McDonough et al. (2015)	High challenge vs. Low challenge Synapse groups; Semantic classification fMRI task	Increased efficiency at modulating brain activity
		14 weeks	
		60–90 yrs	
iPad training	Chan et al. (2014)	iPad tablet computer training	Better episodic memory
		12 weeks	Faster processing speed
		60–90 yrs	
Senior odyssey	Stine-Morrow et al. (2008)	Group-based problem solving of ill-defined problems vs. no-treatment control	Faster processing speed Better inductive reasoning
		20 weeks	Better divergent thinking
		58–93 yrs	
Experience corps	Carlson et al. (2008)	Mentoring elementary school students	Better episodic memory
		Academic year	Improved executive functioning
		60 + yrs	
Experience corps fMRI	Carlson et al. (2009)	Mentoring elementary school students	Improved executive functioning (attentional inhibitory control)
		Flanker task	Increased left prefrontal cortex activity
		6 months	Increased anterior cingulate cortex activity
		60 + yrs	
Aerobic exercise	Colcombe et al. (2006)	Aerobic training versus stretching and toning control	Gray matter increases White matter increases
		6 months	
		60–79 yrs	
Exergame training	Adcock et al. (2020)	Step-based cognitive games, dancing, and Tai Chi vs. control group	Better working memory Improved inhibition
		16 weeks	
		65 + yrs	
Method of loci training	Engvig et al. (2010)	Method of Loci mnemonic training vs. passive control	Better episodic source memory Cortical thickness increases
		8 weeks	
		42–77 yrs	
Juggling training	Boyke et al. (2008)	3-ball cascade juggling vs. control group	Gray matter increases
		3 months	
		50–67 yrs	
Spatial navigation training	Lovden et al. (2012)	Virtual environment spatial navigation training with treadmill vs. treadmill control	Reduced hippocampal shrinking Improved navigation
		4 months	
		20–30 yrs and 60–70 yrs	
Cognitive app training	Bonnechere et al. (2021)	Smartphone/tablet app training	Faster processing speed
		100 sessions	
		60–80 + yrs	

This table is not intended to be an exhaustive list.

To experimentally manipulate busyness, one group of participants could be required to engage in a certain number of tasks for a specified duration/frequency. Pilot studies could determine the optimal number of activities to require, by video-tracking or detailed logging of pilot participants’ busyness/activity levels.

The key would be to allow participants to choose which activities they perform to keep themselves busy, rather than prescribing activities. Thus, the intervention would manipulate the busyness of the individuals rather than the exact type of tasks. Many prior intervention studies understandably focus on specific tasks, such as exercise (see Gomes-Osman et al., 2018), volunteering (Musick et al., 1999), or student mentoring (Carlson et al., 2008). Leaving the choice of the activities up to the participants may reduce stress, as enjoyment would likely be higher for self-chosen activities.

The control group may best be designed as a wait-list control group, where participants eventually receive the option to bolster their busyness levels once the control period is complete. A wait-list design would additionally allow researchers to perform analyses within-participants, when the same individual leads a less versus more busy lifestyle. Cognitive abilities would be assessed pre- and post-intervention and compared between the experimental busy group to the non-busy control group/condition.

### Interaction between busyness, cognition, and stress

**FIGURE 1:**
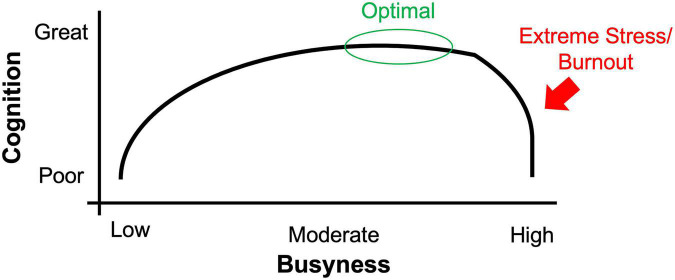
Hypothesized inverted-U relationship between busyness and cognition. Additional research is needed to test this proposed relationship. Optimal cognitive performance is predicted with moderate-to-high busyness, before extreme stress/burnout is reached.

It will also be informative to simultaneously track busyness and stress levels within the same individuals. One individual may find their busy schedule stressful, whereas another may find it enjoyable and fulfilling. Thus, assessing both stress and busyness levels would help determine if busyness is only beneficial if it does not result in a stress response. Indeed, the relationship between busyness and cognition may follow an inverted-U pattern (Yerkes and Dodson, 1908), where moderate levels of busyness are best. See Figure 1. One study observed that, in undergraduates, busier participants also reported more stress (Ramsdell and Festini, 2021). Additional work is needed to systematically evaluate the relationship between busyness and stress as it relates to cognition throughout the adult lifespan.

Another aspect worthy of investigation is whether people enjoy the activities that are keeping them busy. One could imagine different types of busy lifestyles–one with activities of their own choosing, and another with obligatory rather than self-selected activities. The type of activities that keep one busy may predict stress. Therefore, future research would benefit from assessing factors such as the enjoyment of, and the type of, activities contributing to busyness.

### Busyness, cognitive reserve, and brain reserve

Research devoted to cognitive and brain reserve often uses education, occupation complexity, and IQ as proxies of reserve (e.g., Speer and Soldan, 2015; Franzmeier et al., 2017), the idea being that those with higher levels of education, more cognitively demanding occupations, and higher mental capacity are better able to cope with age-related brain pathology (e.g., Stern, 2002; Richards and Deary, 2005). It may be worthwhile to include assessments of busyness in evaluations of cognitive reserve, as busyness may promote cognitive resources similarly to the existing proxies. For instance, occupation complexity is often coded based on the degree to which one’s job requires complex interactions with data (analyzing), people (mentoring), or things (precision working) (Smart et al., 2014). In a similar vein, greater busyness is likely to provide more frequent opportunities for complex daily interactions and new learning, which have been shown to be beneficial (Park et al., 2014; Shors, 2014). Future research could include busyness as a proxy of cognitive reserve, either in isolation, or in conjunction with other measures, as it can provide another window into the complexity of one’s life.

### Longitudinal assessments of busyness and cognition

Just as influential research has evaluated longitudinal changes in both activity levels and cognition, it may similarly be useful to assess longitudinal changes in busyness and cognition. New or existing longitudinal studies that track cognitive ability or conversion to dementia could add a busyness assessment to determine if there are changes in busyness and cognition across the lifespan within the same individuals. Such longitudinal research is also informative for determining how much variability there is in busyness within an individual over the course of their life. The busiest younger adults may similarly be the busiest older adults. Further, it may be that busyness in young- or middle-adulthood is more beneficial at promoting cognitive reserve and resilience in older age. Such questions remain to be evaluated.

### Methodological considerations for the assessment of busyness

#### Ecological momentary assessments

Ecological momentary assessments (EMAs) offer another promising direction for future research on busyness. Instead of asking people to reflect back, EMAs ask participants to answer questions at the present moment, while they are living their normal daily lives (Shiffman et al., 2008). For example, EMAs ask research participants periodically throughout the day to record what they were doing at that moment. This would provide more quantifiable data regarding the number of tasks that people engage in, as well as the relative proportion of time that was spent during different types of activities. One benefit of EMAs is that they are less prone to recall errors (Shiffman et al., 2008), and would provide more ecologically valid measures of busyness. Kamarck et al. (2007) demonstrated that real-life EMAs of job strain collected at 45-min intervals for 6 days predicted future carotid artery blockage, whereas a global questionnaire did not, providing evidence for the superiority of real-time measurements. Further, EMAs of cognitive abilities, like working memory, have been found to be reliable (Sliwinski et al., 2018), demonstrating the option to assess both busyness and cognition using EMAs in real-life settings.

#### Additional survey development

While the MPED (Martin and Park, 2003) is a useful tool, it would be beneficial to develop alternate validated self-report assessments of busyness that measure enjoyment of activities, number of different simultaneous obligations, and organized as opposed to rushed busyness. One could imagine a busy life that is scheduled and organized, still with many tasks and obligations, being different than a frantic and hectic busy schedule. Busyness could also be evaluated at different time frames, such as currently, during the last year, etc.

### Busyness and age

#### Busyness in younger adults

Given the paucity of research on busyness in general, it is not surprising that little research has assessed busyness in younger adults. One study observed that undergraduates who were more academically engaged also tended to be more socially engaged, but there was no significant relationship between episodic memory and academic engagement, social engagement, or busyness (Ramsdell and Festini, 2021). It is likely that the relationship between busyness and cognition is weaker in younger than older adults. Several factors contribute to this hypothesis. First, evidence indicates that, on average, younger adults (ages 20–39) live busier lifestyles than older adults (Festini et al., 2019). Further, on average, younger adults have superior cognitive abilities (Park et al., 2002). Thus, the more limited variability in busyness and cognition, and the lower likelihood of neurocognitive decline in younger adults, makes it less likely that strong relationships will exist between busyness and cognition in younger adults.

#### Busyness and parenthood

Future research should examine differences in busyness for parents versus non-parents. Childrearing adds many daily responsibilities that likely influence busyness levels. It would be worthwhile to evaluate busyness in working and stay-at-home parents and non-parents, including assessment of potential gender differences. Prior research indicates that women tend to be busier (Festini et al., 2019), and, although parent-status was not measured, interestingly, women reported high levels of busyness in the 20s and 30s, whereas men reported *low* busyness in the 20s but high busyness in the 30s, potentially influenced by parenthood (see Verbrugge et al., 1996).

#### Busyness in older adults

Indeed, the potential beneficial effects of a busy lifestyle are likely most noticeable in older adults, who report lower levels of busyness in general (Festini et al., 2019), and have higher likelihood of cognitive decline due to normal aging or underlying pathology (e.g., McDonough et al., 2020). The beneficial effects of a busy lifestyle may also have the largest impact on the wellbeing of older adults and their families, as finding ways to postpone cognitive decline has truly meaningful impacts.

## Discussion and conclusion

Overall, future research on busyness can target many unanswered questions. One critical question will be to test a causal mechanism with an experimental busyness intervention. It will also be valuable to develop additional tools to assess busyness, including EMAs, to measure both busyness and stress within-individuals, and to track busyness longitudinally. Living a busy, yet low stress, lifestyle may be one strategy to boost brain health and is a worthy avenue for additional research.

## Author contributions

SF contributed to the conception of the manuscript, performed the literature review, and wrote and edited all sections of the manuscript.

## Footnotes

1 Forced retirement has causality implications too.
